# Impact of the New Cooperative Medical Scheme on the Rural Residents’ Hospitalization Medical Expenses: A Five-Year Survey Study for the Jiangxi Province in China

**DOI:** 10.3390/ijerph15071368

**Published:** 2018-06-29

**Authors:** Fei Xie, Xiaoqing Jiang, Fang Yuan, Xiaoyun Chen, Zhaokang Yuan, Yuanan Lu

**Affiliations:** 1 Jiangxi Province Key Laboratory of Preventive Medicine, School of Public Health, Nanchang University, Nanchang 330006, Jiangxi, China; 15870030672@163.com (F.X.); tongxue1123@126.com (X.J.); 2 Department of Public Health Sciences, University of Hawaii at Mānoa, Honolulu, HI 96822, USA; fangy@hawaii.edu; 3 High School Affiliated to Fudan University, Yangpu District, Shanghai 200433, China; Shannonv1207@gmail.com

**Keywords:** New Cooperative Medical Scheme (NCMS), medical expense, survey, China

## Abstract

This survey study was conducted to understand the effect of the New Cooperative Medical Scheme (NCMS) on farmers’ medical expenses through comparing the information from five investigations and to obtain a scientific basis for a more applicable NCMS. The survey was carried out through interviewing farmers in their homes. The multi-phase stratified cluster random sampling was adopted to select 3 counties from all 92 counties of the Jiangxi province, 9 townships from the 3 selected counties, 27 villages from the selected 9 townships, and 60 families from each village between 2006 and 2014, and a longitudinal comparative analysis was conducted. The numbers of households/overall sample for the five years were 1924/8082, 1879/8015, 1885/7506, 1890/7857, and 1896/7811, respectively. We collected family members’ social demographic characteristics, health resources, and peoples’ health and medical expenses and reimbursement of each family member. The adjusted hospitalization expenses per capita of township hospitals and county hospitals were totally on a rising trend. However, the costs of tertiary hospitals were on a decreasing tendency. In addition, the expenses for county hospitalization per admission were on an upward trend in general. Furthermore, the total hospitalization expenses and reimbursement per capita (the insurance paid out for the hospitalization expenses) were also all on an upward trend. The proportion of reimbursement also had a tendency of increasing from 24.41% in 2006 to 41.34% in 2014. The costs paid from farmers’ pockets were fluctuated, but in general all lower than the costs in 2006. Furthermore, the percentage of hospitalization expenses from farmers’ annual incomes gradually decreased each year from 56.38% in 2006 to 26.58% in 2014. NCMS program has had an obvious impact on the hospitalization expenses in the Jiangxi rural area. It reduced the hospitalization expenses of the tertiary hospitals significantly. In addition, the program has also encouraged farmers to get more health care. However, there are still some shortages associated with present construction of the NCMS. Hence, there is a need for local government to continue to take effective countermeasures to control the rising trend of hospitalization expense.

## 1. Introduction

The New Cooperative Medical Scheme (NCMS) plays a critical role in protecting farmers’ health: more than 760 million rural residents were covered by NCMS in China in 2016. The goal of the NCMS is to reduce the catastrophic medical expenditure of farmers and to improve the health status of farmers.

In the 1980s, the failure of community based cooperative economy led to a collapse of the Cooperative Medical Scheme: less than 5% of farmers accessed health insurance in the late 1990s and the direct result was that farmers lost their health insurance and were exposed to heavy disease burdens [1]. The 2003 National Health Survey found that over 30% of rural residents did not seek for health care when they were ill and 40% of those individuals thought that the medical cost was too high to pay to them. 

The government managed to implement the New Cooperative Medical Scheme, which was based on the traditional Cooperative Medical Scheme, to alleviate this situation in 2003 [2,3]. The funding source of NCMS was from farmers, local government, and central government. Under the current NCMS premium payment plan, the central and local governments each pays a subsidy of 10 Renminbi (RMB, Chinese currency) per beneficiary per year, while the individuals were required to pay another 10 RMB, meaning that for each NCMS enrollee, a total sum of 30 RMB was contributed to the fund, and the risk pooling is based on the county level [4]. Meanwhile, the average income per capita and the average health expense per capita of rural resident was 2622.2 RMB and 274.67 RMB, respectively in 2003 [3]. The reimbursement covered general diagnosis and treatment expenses (procedure charges, doctor’s service charges, nurse service charges, injection charges) and drugs’ charges and the use of medical instruments charges.

Farmers were enthusiastic about the participation of the NCMS and the satisfaction to the NCMS was also maintained at a high level [5,6,7]. Financial subsidies from the government greatly mobilized the enthusiasm of farmers to participate in the NCMS, and the government has also raised its subsidy standards greatly to cope with the demand of the explosive growth of insured farmers year by year.

In 2014, the financial subsidies per person from government have risen to 370 RMB, and insurance expense from individuals was 70 RMB. The reimbursement rate for hospitalization in Jiangxi province at designated medical institutions varied among different levels of hospitals, with townships at 60%, counties at 40%, and tertiary hospital care center at 30% in 2003 increasing to 90, 80, and 50%, respectively in 2014 (township and county hospitals are located in rural areas, but tertiary hospital care centers are usually located in the centers of urban districts and provide better health care than township and country hospital). The government increased the insurance starting line from 300 RMB in 2003 to 800 RMB in 2008 to 1500 RMB in 2014 [2].

Several studies have reported that patients who had participated in the NCMS were more likely to look for health care services [8,9,10]. Furthermore, a recent study based on a national sample of rural residents from the 2011–2012 wave of China Health and Retirement Longitudinal Study (CHARLS) has shown that the coverage of NCMS associated with less delayed inpatient care [11]. Sun and his co-workers have recently demonstrated that the NCMS reduced the number of medically impoverished households by 24.6% in Yanbian [12]. Rural residents have greatly benefited from the implementation of the NCMS [13].

Did the NCMS really reduce health expenditure of farmers? Another recent report has shown that financial difficulties are not the major causes of the delay for health care service demand but NCMS has reduced the burdens of farmers’ medical expenses to a certain extent [14]. Wang’s team studied the medical expenditure of farmers for certain diseases and revealed that the NCMS has some impacts on reducing the catastrophic medical expenses [15]. However, opposing conclusions also exist in the papers from several groups [16,17,18,19,20]. Recently, a published paper has shown that catastrophic health expenditure and health payment-induced poverty have little to do with the implementation of the NCMS reimbursement since there is no significant difference between before and after of the NCMS [21]. This result is contrary to the current understanding that the NCMS effectively reduced the burdens of medical expenses for farmers.

Since previous reports have not considered the impact of changes in inflation rates on hospitalization costs, this present study has focused on the impact of this factor and exploring whether NCMS has reduced the hospitalization costs of farmers in the Jiangxi province and alleviated the medical burdens of farmers. Moreover, this survey was conducted to clarify whether NCMS encourages farmers to change their choice of hospital.

## 2. Materials and Methods

### 2.1. Methods

The survey was started in the Jiangxi province in 2006 and continued in 2008, 2010, 2012 and 2014. As well, the survey was conducted through a multistage stratified random cluster sampling method. First, according to the basis of the sampling method from the Ministry of Health of the People’s Republic of China, all the counties of Jiangxi Province were ranked basing on the farmers’ average incomes, and then they were divided into three levels by the percentile method (<33.33%, 33.33–66.67%, and >66.67%). Next, we chose three counties (Xiushui, Wuyuan, and Luxi) to represent the different economic levels. Then, we utilized the percentile method according to each township’s agricultural population to choose three townships from each county. Furthermore, 27 villages were extracted from the selected townships through the same way. Finally, we almost interviewed all the households in the townships. The numbers of households/overall sample for the five years were 1924/8082, 1879/8015, 1885/7506, 1890/7857, and 1896/7811, respectively. In this survey, all the people who registered in the residence booklet in the household and those who lived in the village for over half a year holding a non-local residence booklet were included in this survey. The unit of this survey to be analyzed was every family member in the household.

### 2.2. Questionnaires and Data Collection

This survey study was conducted basing on the questionnaires from the Ministry of Health of China with minor modification in order to conform to the social condition of Jiangxi Province. The questionnaires included family members’ social demographic characteristics, questions about health resources, and peoples’ health and medical expenses and reimbursement. With the help of staff from the local government, we conducted this survey through a ‘one-to-one interview’, pattern, which means one investigator interviewed one respondent. The investigators were undergraduate and graduate students from the School of Public Health at Nanchang University, and the interpreters were the local government staffs. Our interview targeted on the head of households because they are more representative and also since most young people went away from their homes for employment. However, other members of the families were interviewed when the house head was not at home at that time. The response rate was nearly 100% under the support of the local government and residents. 

### 2.3. Quality of Data

Before conducting this survey, the investigators were trained centrally by public health Professor Yuan, who has extensive knowledge and experience in survey studies. In particular, all the questionnaires were carefully evaluated and analyzed to ensure that there was no unclear question. Furthermore, we reviewed every questionnaire at night and phoned the respondents if there was any information missing or unclear. We used χ² tests among the respondents’ different demographic characteristics, and the standard was 0.05 for all tests. EpiData 3.0 software (The EpiData Association, Odense, Denmark) was used for data entry and the IBM SPSS 19.0 software package (IBM Corp., Armonk, NY, United Sates) was used for data analysis.

In addition, the Myers blended index was also introduced in this study to estimate the quality of the survey data. The Myers blended index is one of the common methods for testing age accumulation. It assumes that there is not any data preference in a population first. Then a number ranging from 0 to 9 at the end of the respondents’ age with should account for one-tenth among the total population. The sum of the absolute values of the differences between the actual population age distribution and the theoretical distribution is called the Myers blended index. The Myers blended index ranges from 0 to 99. We calculated the distribution of the age of respondents by every 10 years and checked the results if it was disproportionate. The Myers blended index should be lower than 60 to ensure the quality of the data [22,23].

### 2.4. Data Analysis

The consumer price index (CPI) which measures changes in the price level of a market basket of consumer goods and services purchased by households was used to calculate the inflation rates in this study. Moreover, medical expenses were adjusted through inflation rates to eliminate the influence of inflation on medical expenses to achieve visual data to better explain whether the NCMS has helped farmers to reduce medical expenses. The CPI, the most reliable data in China, was obtained from the China health statistics yearbook. According to the formula from the book of *China Statistical Yearbook*, we calculated the inflation rates for every year from 2006 to 2014, and the price of 2006 was used as the baseline.

Inflation Rate of Year x + 1 = 1 × (1 + CPI of Year x + 1) − 1(1)

Inflation Rate of Year x + 2 = 1 × (1 + CPI of Year x + 1) × (1 + CPI of Year x + 2) − 1(2)

In addition, we used the inflation rate to modify the original data.

Adjusted data = Original data / (1 + CPI)(3)

Finally, the adjusted data for different years were compared to figure out if NCMS has had an impact on farmers’ medical expenses.

## 3. Results

### 3.1. Myers Blended Index

In this study, the Myers blended indexes of five surveys were 5.12, 12.02, 8.52, 3.54, and 7.07, respectively. As shown in Table 1, we use the data from 2014 to be an example to show how we get Myers blended indexes. They were all lower than 60, meaning that the data was sufficient with good quality.

### 3.2. Family Members’ Social Demographic Characteristics

As shown in Table 2, the numbers of households/overall sample for the five years were 1924/8082, 1879/8015, 1885/7506, 1890/7857, and 1896/7811, respectively. The average number of family members per household for the five years were 4.20, 4.27, 3.98, 4.16, and 4.12, respectively. The men/women ratios were 1.05, 1.05, 1.13, 1.07, and 1.08, and the average age of all the participants were 34.26, 34.85, 35.54, 35.46, and 36.60 years old, respectively. The number of hospitalizations were 129, 304, 429, 628, and 747, respectively. Besides the average length of stay in hospital were 13.21, 9.55, 9.75, 11.40, and 11.77 days, respectively. The *χ*^2^ tests did not show any significant difference by gender across waves (χ^2^ = 6.192, *p* > 0.05). However, the analysis of the participant’s age showed that there was a significant statistical difference (*p* < 0.001).

### 3.3. Analysis of Hospitalization Medical Expenses

**Figure 1:**
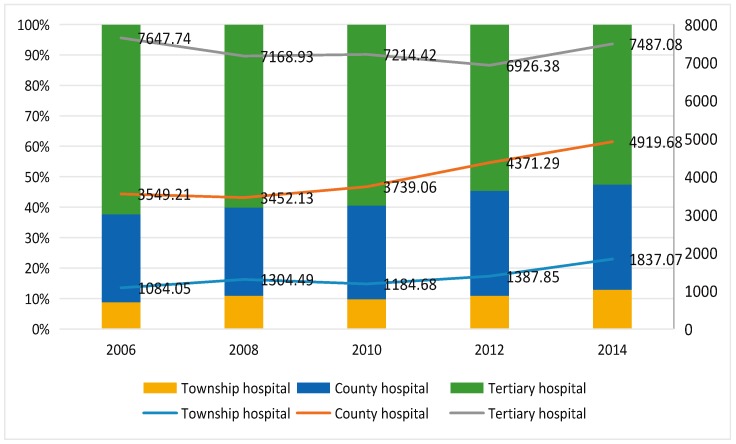
The adjusted hospitalization expense per capita in different levels of medical institutions and the distribution (¥). The lines show the adjusted data of hospitalization expense per capita in different levels of medical institutions. The bar chart shows the distribution of the proportion of each level hospital expense in total expense.

Figure 1 showed the hospitalization expense per capita in different levels of medical institutions with the line and used the bar graph to represent the percentage of medical expenses in different medical institutions. The study showed that the adjusted hospitalization expenses per capita of township hospitals and county hospitals were rising slowly in general, rising from 1084.05 RMB per capita to 1837.07 RMB per capita and 3549.21 RMB per capita to 4919.68 RMB per capita, respectively. Moreover, adjusted expenses of the tertiary hospitals (those above city and county) were on a decreasing tendency since 2006 except for year 2014. The results above indicated that the NCMS had controlled the increasing medical costs effectively. In this study, medical expenses in township hospitals and county level hospitals were taking a larger proportion substantially and the proportion of tertiary hospital decreased from 62.27% to 52.56%, which means farmers were more likely to go for a doctor more nearby their villages. 

**Figure 2:**
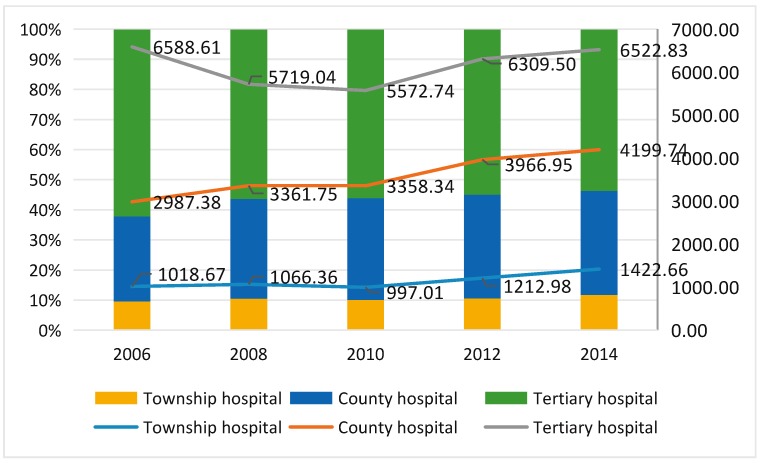
The adjusted hospitalization expense per admission in different levels of medical institutions and distribution (¥). The lines show the adjusted data of hospitalization expense per admission in different levels of medical institutions. The bar chart shows the distribution of the proportion of each level hospital expense in total expense.

The adjusted expenses for county level hospitalization per admission were on an upward trend, increased from 2987.38 RMB per admission in 2006 to 4199.74 RMB per admission in 2014. However, the inpatients’ medical expenses per admission at township hospitals and tertiary hospitals all declined at first and then increased after correcting with CPI, and the year of 2010 was the turning point time. Moreover, the adjusted hospitalization cost at tertiary hospitals in 2006 was the highest. Similarly to hospitalization expenses per capita, the proportion of inpatients’ medical costs at township and county level hospitals was rising firmly from 9.61% in 2006 to 11.71% in 2014 and from 28.20% in 2006 to 34.58% in 2014, respectively. However, the respective proportion from tertiary hospitals declined from 62.19% in 2006 to 53.71% in 2014 (Figure 2).

**Figure 3:**
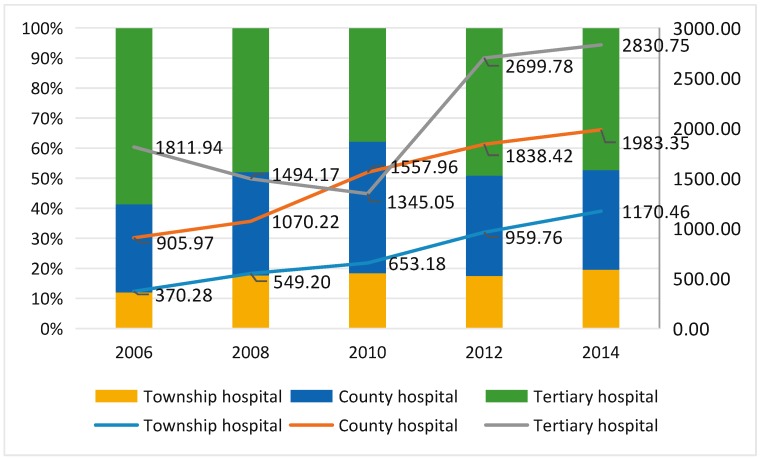
The reimbursement per capita in different levels of medical institutions (¥). The lines show the adjusted data of reimbursement in different levels of medical institutions. The bar chart shows the distribution of the proportion of each level reimbursement in total expense.

As shown in Figure 3, the adjusted reimbursement per capita at township level and county level medical hospitals were all in an upward trend in general. The amount of adjusted reimbursement from township institutions was increased to 1170.46 RMB per capita in 2014 as compared to 370.28 RMB per capita in 2006, showing an increase by more than three times. The reimbursement per capita from county hospitals was also increased two-fold in 2014 as compared to that in 2006. However, the reimbursement per capita amount from tertiary hospitals adjusted with CPI showed a sharp decrease from 1811.94 RMB per capita in 2006 to 1345.05 RMB per capita in 2010 first and then increased to 2830.75 RMB per capita in 2014, showing a sharp turn in this changing process. In general, the adjusted amount of increased reimbursement from tertiary hospitals was the least among the three hospital levels.

As shown in Table 3, the adjusted total hospitalization expenses and reimbursement per capita were all in an upward trend. Furthermore, the proportion of reimbursement in hospitalization expenses had the tendency of increasing similarly from 24.41% in 2006 to 41.34% in 2014. As for the adjusted costs paid from the farmers decreased from 2639.74 RMB per capita in 2006 to 2041.85 RMB per capita in 2008 and then rose to 3357.12 RMB per capita in 2014. In addition, the adjusted costs paid by farmers were fluctuated, but they were all lower than the costs in 2006. Furthermore, the percentage of hospitalization expenses paid by farmers in their annual incomes was decreasing each year, from 56.38% in 2006 to 26.85% in 2014.

## 4. Discussion

### 4.1. Per Capita and Secondary Hospitalization Expenses

The results of this study have shown that the hospitalization expenses per capita and per admission in township and county hospitals have been rising, particularly for county hospitals. However, at tertiary hospitals, the per capita and per admission costs for hospitalization are fluctuating and even appear to decline. This may reflect the change of farmers in their decision to seek for health care, indicating the positive impact of the NCMS that more farmers were encouraged to utilize medical institutions at township and county levels. We speculate that the change in the NCMS reimbursement ratio affected the farmers’ choice of hospitals, especially for tertiary hospitals. Besides, we cannot ignore the effects from the increase in the use of outpatient primary care and preventive services, which helped to decrease the use of hospitalizations. NCMS gradually increased the hospitalization reimbursement proportion for township and county-level hospitals, in addition of decreased amount for deductible hospitalization payment. At the same time, tertiary hospitals have also decreased the reimbursement and down payment portion for hospitalization. These initiatives encourage farmers to select township and county-level medical institutions since they are likely to receive more reimbursement and their own medical expenses will be lowered. Because township hospitals are generally smaller than county-level hospitals, farmers seek better medical service at county hospitals. This caused the average hospitalization expenses per capita and per admission to increase more at county hospitals than at township hospitals.

Furthermore, most interviewed subjects in this study were farmers and their annual income is generally low. Therefore, whenever they get sick, they usually try to perform self-diagnosis according their past medical experience and purchasing cheap medications for treatment in to reduce medical expenses [24]. However, findings from this survey study show that the farmers’ per capita and per admission hospitalization costs for farmers was actually increased. This indicated that the NCMS program has impacted farmers’ necessary demand for medical services. More importantly, implementation of the NCMS program has increased their personal view on their own health.

### 4.2. Reimbursement Costs

This study shows that although reimbursement costs and ratios fluctuate in amount, there is a rising general trend. This reveals how NRCMS plays a meaningful role in alleviating farmers’ burden on medical expenses. Figure 3 shows that in 2010, reimbursement amount for tertiary hospitals reduced substantially, followed by an increase. This was due to the government’s adjustment on tertiary hospitals’ reimbursement system at the end of 2008 [25]. The government increase the insurance starting line to 800 RMB. To a large extent, this new policy led farmers to select hospitals that are of lower levels than tertiary hospitals.

### 4.3. Reimbursement of Total Medical Expenditure and Rural Residents’ Cash Expenditure on Medical Expenses

This study revealed that the average amount of medical expenses paid by farmer themselves in 2008, 2010, 2012, and 2014 are lower than in 2006. Also, hospitalization expenditure takes up a smaller percentage in farmers’ annual salaries year by year, showing the increase in hospitalization costs is now under good control of the NCMS program. This finding is the same as the report from Yang and coworkers [26]. However, there are still some studies with different results stating that farmers’ burden on their medical service expenditure remains heavy [27,28]. It should be noted that some of these studies did not exclude the effects from CPI, causing that the hospitalization expenses remain to rise rapidly. Some other studies only analyzed expenditures on some specific diseases. In sum, the increases in expenditures for a specific disease does not mean an increase for the overall medical expenditures for all situations, as supported by Jing S. et al.’s report [29]. Other studies categorized farmers into poverty and non-poverty subgroups and discovered that NCMS has no obvious effect on the medical expenses for farmers suffering poverty. However, in Yu et al.’s study [30], it reveals that NCMS did lower farmers’ medical expenditure even though they are in poverty. Taken together, NCMS decreased medical expenses overall: which is consistent to this study’s results. In addition, present study did not categorize farmers into different subgroups and thus further research needs to focus more on analyzing different farmer groups based on their annual incomes for their medical expenses with other perspectives.

The study’s data also reveal that reimbursement proportions are increasing year by year, revealing that NCMS is constantly improving to ensure that farmers with low income could still receive effective medical services. However, the reimbursement ratio for 2014 was 41.34%, which is far from the three level hospitals average reimbursement rate 73.33%. This is primarily due to a large category of clinical drugs not being included for reimbursement by NCMS and also some doctors working with their partnered drug suppliers attempted to increase the price of those reimbursable drugs and so they can make profit from farmer patients [31,32].

In conclusion, NCMS has enabled farmers to reduce the amount of their annual hospitalization expenditures, which is a meaningful accomplishment in reducing farmers’ medical burden. More in-depth studies should be designed to advance present studies to other areas of this field, such as by further analyzing hospitalization costs, examining outpatient medical expenses, analyzing the flow of the treatment process, and categorizing farmers into different subgroups.

## 5. Limitation

In our study, though we can get most of the medical expenses from the special pamphlet of NCMS of farmers, there are still some farmers who cannot find their pamphlet and we had to collect the data through their recall. Therefore, we cannot avoid the recall bias completely. However, all the farmers responded to the questionnaires so that we avoided the non-response bias perfectly with the help of local government staffs. Meanwhile, the help may cause a selection bias, but we communicate with farmers face to face and one to one. Therefore, it may not be an important issue.

This study focused only on the hospitalization expenses without considering the outpatients’ expenses. The reimbursements of outpatients will feedback in one week so that we cannot get most of the reimbursement of outpatients at once. Therefore, we cannot express the whole medical expenses except the hospitalization expenses and also we cannot exclude the number or complexity of procedures and presence of comorbidities performed on patients. In addition, we did not divide farmers into different groups to describe the degree and difference of impact of NCMS policy on these different farmers. Furthermore, though we assessed the hospitalization expenses, this study did not discuss the impact of NCMS on farmers’ health and also we are unable to determine whether the quality of farmers’ health care becomes improved or not. Additionally, some recent studies have revealed that over-prescribing antibiotics is very common in rural areas and quite a lot of the village clinic doctors have not passed their medical exams to become certified physicians [32,33].

Most of the farmers were living in the same village in these years, however, some of the farmers would go to the cities or other places to find some extra jobs to earn more money. Thus, we cannot be sure that we interview the same people every year. Therefore, the potential migratory flow in China should be taken into account. Nevertheless, this study was done in the same villages and got the residence booklets from the households; therefore, the mentioned risk has been minimized.

## 6. Conclusions

NCMS has an important impact on hospitalization expenses in the Jiangxi rural area. This medical program has reduced the hospitalization expenses of the tertiary hospitals significantly. In addition, the proportion that hospitalization expenses accounted for the annual incomes of every farmer was in a decreasing trend. Furthermore, the program encourages farmers to get more necessary health care rather than diagnosing disease by themselves. However, there were still some limitations in the construction of current NCMS, such as lots of drugs not being included for reimbursement. Hence, the government should continue to improve NCMS by controlling the rising trend of hospitalization expenses.

## Figures and Tables

**Table 1:** Myers blended index of Jiangxi province in 2014.

The Ending Figure of Age	10–49 Years Old			20–59 Years Old			(4) + (7)	Percentile	ǀ(9)−10ǀ ^c^
	NO.	Weights	(2) × (3) ^a^	NO.	Weights	(5) × (6)		(8)/45039 ^b^	
(1)	(2)	(3)	(4)	(5)	(6)	(7)	(8)	(9)	(10)
0	390	1	390	419	9	3771	4161	9.24	0.76
1	377	2	754	426	8	3408	4162	9.24	0.76
2	424	3	1272	456	7	3192	4464	9.91	0.09
3	376	4	1504	361	6	2166	3670	8.15	1.85
4	444	5	2220	482	5	2410	4630	10.28	0.28
5	518	6	3108	530	4	2120	5228	11.61	1.61
6	461	7	3227	480	3	1440	4667	10.36	0.36
7	464	8	3712	528	2	1056	4768	10.59	0.59
8	478	9	4302	517	1	517	4819	10.70	0.70
9	447	10	4470	477	0	0	4470	9.92	0.08
Total							45039	100.00	7.07

^a^ The third line with number 1 to 10 was the order of the column and we calculate by the order in the second line; ^b^ The eighth line divided by the total of (8); ^c^ The absolute value of the ninth line minus 10.

**Table 2:** Demographic characteristics of rural residents in Jiangxi province.

Variables	2006	2008	2010	2012	2014
Number of interviewees	8082	8015	7506	7857	7811
Number of households	1924	1879	1885	1890	1896
Average number of family members per household	4.20	4.27	3.98	4.16	4.12
Gender ^a^					
Male	4144	4102	3974	4073	4054
Female	3938	3913	3532	3784	3757
Female/Male	1.05	1.05	1.13	1.07	1.08
Age (%) ^b^					
<5	5.88	6.38	6.59	7.32	5.94
5–	9.63	10.23	11.02	12.13	14.15
15–	20.18	17.40	15.07	12.98	9.85
25–	18.30	18.35	17.03	17.13	16.68
35–	16.56	15.93	16.39	15.46	15.47
45–	13.02	13.14	14.65	14.70	15.76
55–	8.64	9.86	11.06	11.79	12.34
65–	7.81	8.71	8.19	8.49	9.82
Average age (year) ^c^	34.26	34.85	35.54	35.46	36.60
Number of hospitalizations	129	304	429	628	747
The average length of stay in hospital (day)	13.21	9.55	9.75	11.40	11.77

^a^ Gender composition inspection: χ^2^ = 6.192, *p* > 0.05; ^b^ The age distribution inspection: *p* < 0.001; ^c^ Average age referred to the entire sample included all the members of every household.

**Table 3:** Adjusted total hospitalization expense and the expense paid by farmers (¥).

Variables	NO.	2006	2008	2010	2012	2014
Hospitalization expense	1	3492.25	3324.33	3289.27	4202.51	4441.76
Reimbursement	2	852.51	961.94	1247.41	1789.29	1836.11
Proportion of reimbursement (%) ^a^	3	24.41	28.94	37.92	42.58	41.34
Paid by farmers	4	2639.74	2362.39	2041.85	2413.22	2605.65
Annual income per capita of farmers ^b^	5	4682.19	5559.91	6560.55	8154.60	9704.24
4/5 (%) ^c^	6	56.38	42.49	31.12	29.59	26.85

^a^ Proportion of reimbursement: the proportion of hospitalization expenses paid by insurance, use the data of number 2 divide by number 1; ^b^ The income data get from the statistical yearbook of Jiangxi province; ^c^ Use the data of number 4 divide by number 5.
